# Plasma membrane effects of sphingolipid-synthesis inhibition by myriocin in CHO cells: a biophysical and lipidomic study

**DOI:** 10.1038/s41598-021-04648-z

**Published:** 2022-01-19

**Authors:** Bingen G. Monasterio, Noemi Jiménez-Rojo, Aritz B. García-Arribas, Howard Riezman, Félix M. Goñi, Alicia Alonso

**Affiliations:** 1 Instituto Biofisika (CSIC, UPV/EHU), Universidad del País Vasco, 48940 Leioa, Spain; 2 Departamento de Bioquímica, Universidad del País Vasco, 48940 Leioa, Spain; 3 NCCR Chemical Biology, Department of Biochemistry, University of Geneva, 1211 Geneva, Switzerland

**Keywords:** Biophysics, Chemical biology

## Abstract

Suppression of a specific gene effect can be achieved by genetic as well as chemical methods. Each approach may hide unexpected drawbacks, usually in the form of side effects. In the present study, the specific inhibitor myriocin was used to block serine palmitoyltransferase (SPT), the first enzyme in the sphingolipid synthetic pathway, in CHO cells. The subsequent biophysical changes in plasma membranes were measured and compared with results obtained with a genetically modified CHO cell line containing a defective SPT (the LY-B cell line). Similar effects were observed with both approaches: sphingomyelin values were markedly decreased in myriocin-treated CHO cells and, in consequence, their membrane molecular order (measured as laurdan general polarization) and mechanical resistance (AFM-measured breakthrough force values) became lower than in the native, non-treated cells. Cells treated with myriocin reacted homeostatically to maintain membrane order, synthesizing more fully saturated and less polyunsaturated GPL than the non-treated ones, although they achieved it only partially, their plasma membranes remaining slightly more fluid and more penetrable than those from the control cells. The good agreement between results obtained with very different tools, such as genetically modified and chemically treated cells, reinforces the use of both methods and demonstrates that both are adequate for their intended use, i.e. the complete and specific inhibition of sphingolipid synthesis in CHO cells, without apparent unexpected effects.

## Introduction

Sphingolipids (SL) are considered as fundamental structural components of biological membranes. They are involved in many important and different biological roles, such as cell proliferation, differentiation, apoptosis, and ageing, as well as embryogenesis^1^. Most SL metabolic reactions are bidirectional, thus metabolites are interconvertible and it is often the specific species ratio what determines the cell fate^2^. Besides, SL metabolic effects vary depending on the cell type, subcellular compartment, cell cycle phase, or extracellular stimulus^3,4^. An example of the complex effects of SL metabolites is given by sphingosine-1-phosphate and ceramide (Cer), the former is a second messenger for cell survival and proliferation, while the latter can induce cell death^5,6^.

Many aspects of SL function in cells remain to be understood. One testable approach to improve our understanding of SL roles consists of examining the effects of SL restriction in cell membranes. Lowering SL concentrations in the growth medium has little effect by itself, since mammalian cells can usually synthesize their own SL. Thus, researchers in this field have attempted to control the first, pacemaker enzyme in the SL synthetic pathway, serine palmitoyltransferase (SPT). This enzyme catalyzes the reaction between palmitoyl-CoA and L-serine to synthesize 3-dehydro-D-sphinganine^7^. Combining SL restriction in the growth medium (usually lowering the concentration of fetal bovine serum, FBS) with annulling SPT activity could lead to a very severe dearth of SL in the cells. SPT activity could be removed following one of at least two strategies, either genetic mutation or chemical inhibition.

As an example of the former approach, using a genetic selection method in CHO^8^, the Hanada lab isolated the mutant LY-B cell line, that exhibited a loss of SPT enzyme function through a defective SPTLC1 subunit. The mutant cells maintained the ability to take up and metabolize exogenous sphingoid bases from the culture medium^8^. LY-B cells have been used in multiple studies exploring SL effects, and SL interaction with glycerophospholipid metabolism^9–12^. Mutant LY-B and wild type CHO cells could be comparatively studied to determine the effect of SL depletion on the cell membrane biophysical properties. In our previous study^13^, with cells grown under limiting SL concentrations, a significant decrease in the rigidity of LY-B cell membranes was observed using laurdan fluorescence, as well as a decrease in membrane breakthrough forces (membrane nanomechanical resistance) assessed by atomic force microscopy (AFM)^13^. Concomitantly, SL concentration in membranes was drastically reduced, with partially compensating changes in glycerophospholipids^13^.

An alternative strategy to detect the effects of an overall SL decrease would be the specific inhibition of the SPT enzyme. The antifungal myriocin has been applied to a variety of studies. In Jurkat acute leukemia cells, myriocin blocks the ceramide de novo synthesis^14^. In particular it inhibits the synthesis of long-chain Cer species which stimulate proteasomal activation with subsequent activation of caspases^15^. Myriocin has also been used for identifying several SPT inhibition effects in membrane structure and functions. For example, the role of skin sphingosine in the permeation of levodopa (a hydrophilic drug) across rat skin^16^, the paradoxical effects on barrier permeability homeostasis^17^ and the delays of mammalian epidermal barrier recovery after acute perturbation^18^ have been studied. In a recent contribution from one of our laboratories, the cellular responses to SL depletion have been described, highlighting the importance of SL in particular pathways such as protein secretion from the endoplasmic reticulum^19^.

In the present study, we explore the effects of myriocin-induced SPT inactivation in CHO cells. In particular, the consequences on cell growth, lipid composition, and membrane physical properties have been considered. A novel plasma membrane (PM) preparation (‘PM patches’) has been used^20,21^. The study has been performed following the steps of the previous publication with genetically modified CHO cells (LY-B cells)^13^, so that the respective results can be compared. The LY-B study design was such that the effects of FBS removal, namely loss of sphingolipids and loss of other components in the serum, could not be properly differentiated. Myriocin allows the specific inhibition of SPT even in the presence of a full complement of FBS. Conversely, our study might unveil myriocin effects other than SPT inhibition.

## Results

### Growth and viability

**Figure 1:**
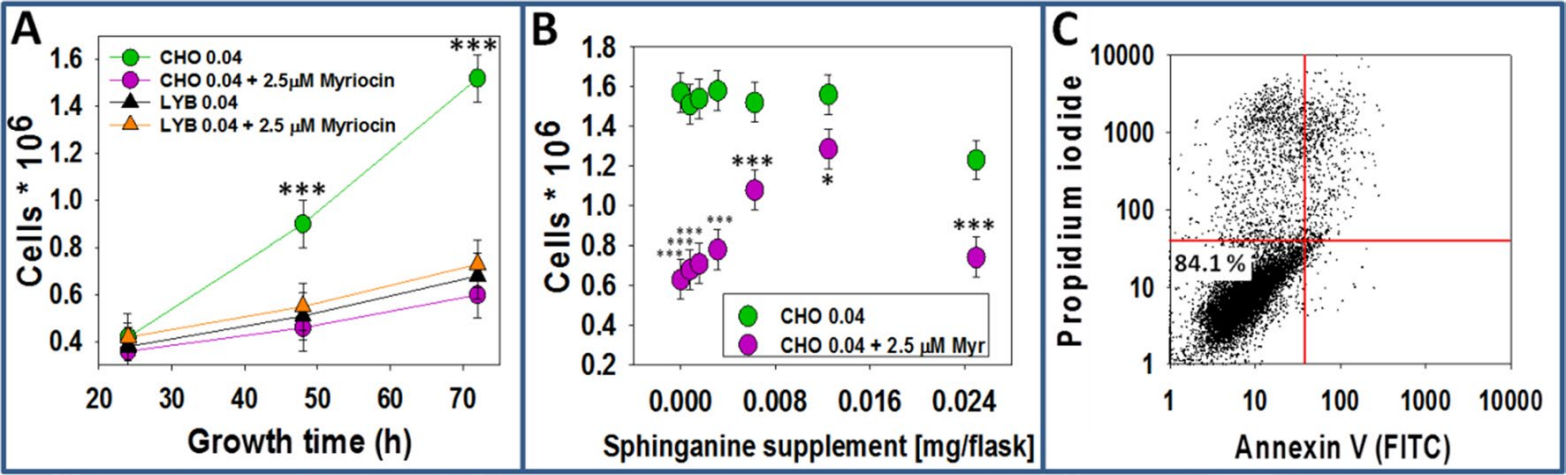
Myriocin-treated CHO and LY-B cell growth. (**A**) LY-B and CHO cell growth as a function of time in sphingolipid-deficient (0.04% FBS) medium, plus/minus 2.5 µM myriocin. Statistical symbols correspond to differences between green circles (CHO 0.04) and the other three samples; Student’s t-test ***P < 0.001. (**B**) Myriocin-treated and non-treated CHO cell growth after 72 h in sphingolipid-deficient medium supplemented with sphinganine (seeded cells: 0.25 × 10^6^). Values are given as average values ± S.D. (n = 3). Significance, according to Student’s t-test, myriocin vs. control: *P < 0.05; ***P < 0.001. (**C**) A representative FACS assessment of cell viability of CHO cells after 72 h in sphingolipid-deficient medium plus 2.5 µM myriocin. Three independent experiments were performed counting 10,000 cells in each case.

The present contribution is devoted to the effects of myriocin on SL synthesis and membrane properties in CHO or LY-B cells. Results are occasionally shown of cells grown in myriocin-free, SL-deficient medium. The latter are only for comparative purposes, they have been published in^13^. Cell count measurements were performed using a BioRad TC20 hemocytometer to assess myriocin treatment effects on CHO (wild type) and LY-B (SPT-defective) cell division rate and integrity. Figure 1A shows a comparison between myriocin-treated and non-treated cell growth rate in SL-deficient (containing 0.04% FBS) medium. Student’s t-test revealed a statistically significant difference between myriocin-treated and non-treated CHO cell division rate; myriocin treatment made CHO cell growth much slower (Fig. 1A). The LY-B cell division rate was equally low in the presence or absence of myriocin. In addition, there was no statistically significant difference between treated CHO cells and non-treated/treated LY-B cells grown in SL-deficient medium. Comparison between cell division rates in standard and deficient medium is shown in Fig. S1A and S1B; myriocin treatment had no effect on the cell division rate when CHO or LY-B cells were grown in standard medium, but some inhibition took place in CHO cells grown on deficient medium (Fig. S1A and S1B).

To ascertain that the poor cell growth at low FBS concentrations was indeed due to the lack of SL, and not to the absence of other nutrients putatively present in FBS, it was tested whether myriocin-treated CHO cells were able to reach the full growth rates when the SL-deficient medium was supplemented with specific SL. For this purpose, equimolar mixtures of egg PC and SM or sphinganine were sonicated in DMEM:F12 medium and added to the culture flasks in various amounts. A good recovery was achieved with sphinganine (Fig. 1B and S1E) or SM (Fig. S1C) supplementation.

Figure 1B shows that myriocin-treated CHO cells grown on SL-deficient medium for 72 h reached ≈ 78% of the control growth when 0.0125 mg sphinganine were added per T25 culture flask (8 µM final concentration), while larger concentrations appeared to have a toxic effect. In the case of SM, 0.1 mg/flask or higher (40 µM or higher) supplementation made myriocin-treated CHO cells reach 75% of the control growth (Fig. S1C), as seen in Monasterio et al*.*^13^. It was concluded that myriocin-mediated lack of SPT activity could be masked when appropriate SL supplementation was added in the deficient-medium.

Next, cell viability was tested performing flow-cytometry analysis of cells stained with Annexin-V-FITC and propidium iodide (Fig. 1C). Flow-cytometry analyses demonstrated that 84% of CHO cells grown in SL-deficient medium for 72 h and treated with 2.5 µM myriocin remained viable (Fig. 1C), similar to the 86% value obtained with non-treated LY-B cells grown in SL-deficient medium^13^. Values for Myr-treated LY-B cells were the same as for the non-treated ones.

#### Lysenin-staining

**Figure 2:**
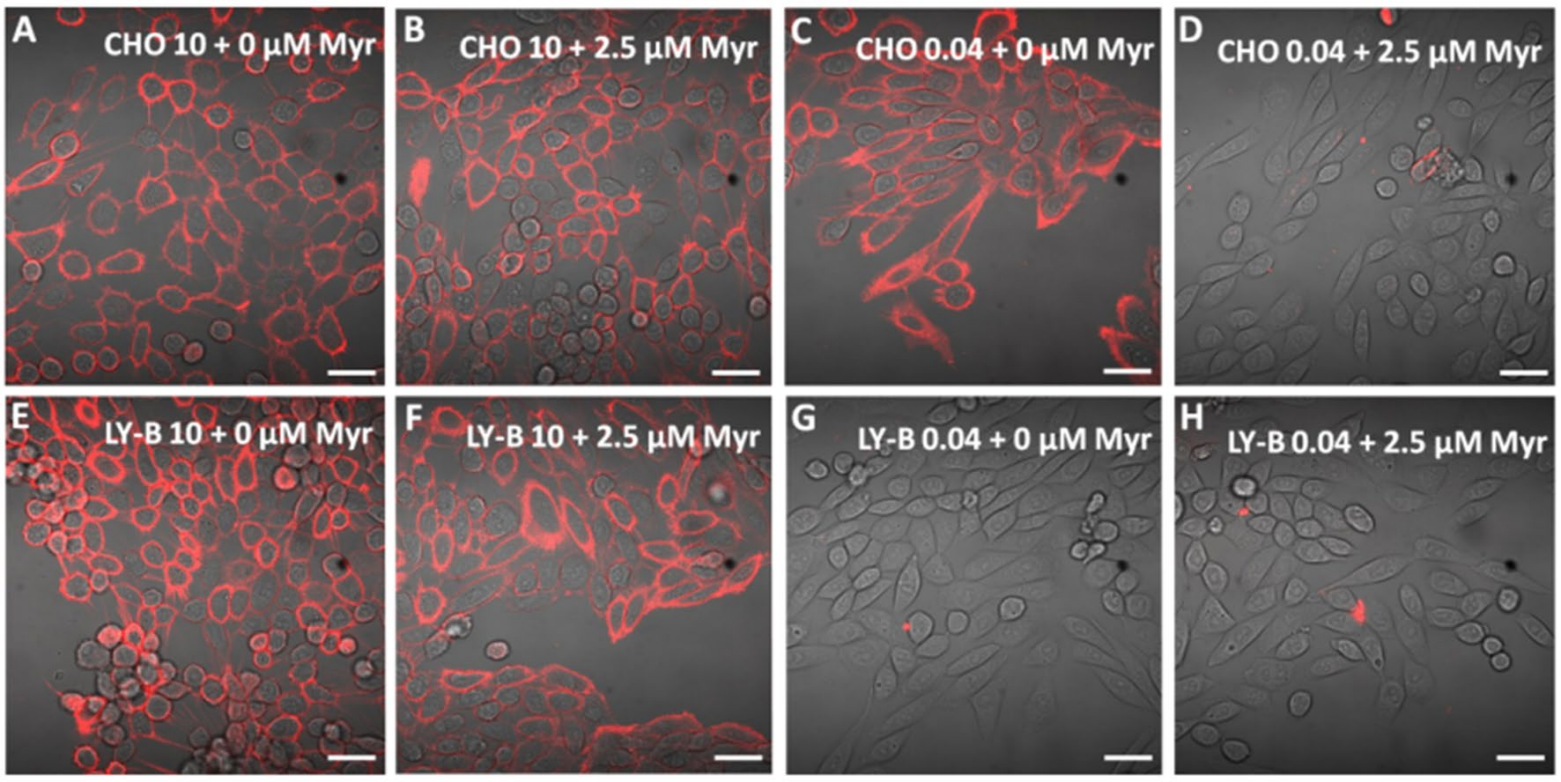
mCherry-Lysenin-stained myriocin-treated or non-treated cells after 72 h growth. Non-treated (**A**) and myriocin-treated (**B**) CHO cells in standard medium. Non-treated (**C**) and myriocin-treated (**D**) CHO cells in deficient medium. Non-treated (**E**) and myriocin-treated (**F**) LY-B cells in standard medium. Non-treated (**G**) and myriocin-treated (**H**) LY-B cells in deficient medium. Bar = 75 µm.

Lysenin is a peptide toxin that binds specifically SM in membranes. A non-toxic fragment of lysenin was expressed as a fusion peptide with mCherry, NT-lysenin-mCherry, as detailed under Methods. Cells were stained with SM-specific NT-lysenin-mCherry, and visualized with confocal microscopy (Fig. 2). Non-myriocin treated CHO cells grown in standard (Fig. 2A) or SL-deficient medium (Fig. 2C), as well as treated CHO cells grown in standard medium (Fig. 2B), appeared thoroughly stained with mCherry. However, only little dots of mCherry were seen in myriocin-treated CHO cells grown in SL-deficient medium (Fig. 1D), indicating a remarkable SM decrease in this sample. In LY-B cells (Fig. 2E–H), the lysenin signal decrease observed in Fig. 2G and H is a direct reflection of their mutated SPT enzyme^13^ and not due to the myriocin treatment, as differences are not seen between non-treated (Fig. 2G) and treated (Fig. 2H) LY-B cells grown in deficient medium.

**Figure 3:**
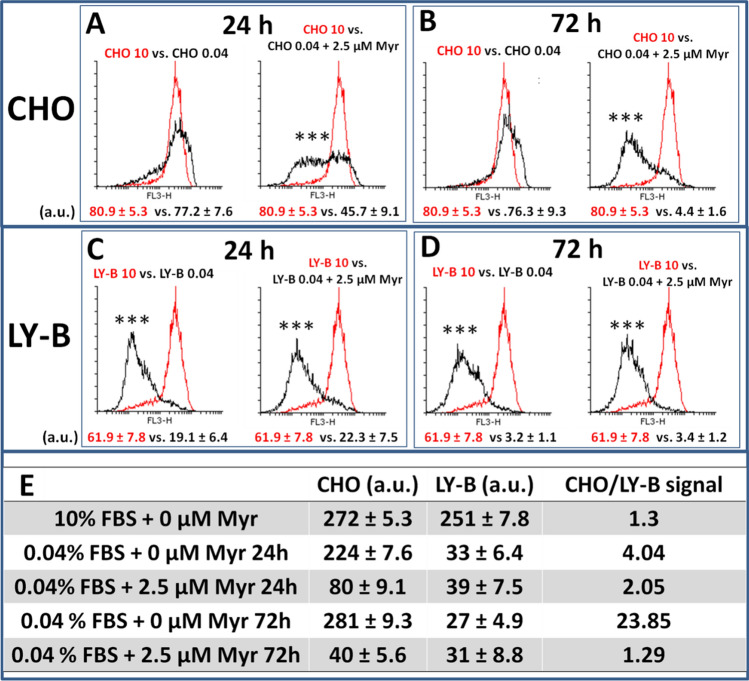
FACS-mediated, quantification of cell staining with SM specific mCherry-lysenin as a function of time. CHO cells grown in standard medium vs. myriocin-treated or non-treated CHO cells grown in deficient medium for 24 h (**A**) or 72 h (**B**). LY-B cells grown in standard medium vs. treated or non-treated LY-B cells grown in deficient medium for 24 h (**C**) or 72 h (**D**). Total CHO and LY-B lysenin-mCherry signals and CHO/LY-B lysenin-mCherry signal ratios (**E**). Histograms in red correspond to control cells (CHO or LY-B grown in standard-medium); histograms in black correspond to the samples of interest, as indicated in each case. Geometric mean ± S.D. (n = 3). Representative histograms are shown; three independent experiments were performed counting 10,000 cells in each case. Statistical significance was calculated with the Student’s t-test, ***P < 0.001.

For lysenin-mCherry quantification, flow-cytometry was performed (Fig. 3). In the first 24 h, CHO cells grown in a deficient medium and treated with 2.5 µM myriocin, underwent a two-fold lysenin signal decrease compared with CHO cells grown in a standard medium (Fig. 3A). On the contrary, there was no statistically significant difference between CHO cells grown in standard medium and non-treated CHO cells grown in deficient medium (Fig. 3A) as seen in Monasterio et al*.*^13^. 72 h were needed to achieve an apparently total SM depletion in myriocin-treated CHO cells grown in deficient medium (Fig. 3B). In the case of LY-B cells, myriocin treatment did not affect the mCherry-lysenin values (Fig. 3C and D), as there was no statistically significant difference between treated and non-treated LY-B cells grown in a deficient medium, neither after 24 h (Fig. 3C), nor after 72 h (Fig. 3D).

For an easier comparison between genetically mutated LY-B and myriocin-treated CHO cells, Fig. 3E shows a table of CHO/LYB signal ratios. The most significant conclusion is that after 24 h myriocin treatment, CHO cells had twice the signal intensity of LY-B, and that the difference was suppressed after 72 h. No statistically significant difference was found between LY-B and myriocin-treated CHO cells at this stage. Results indicate, as expected, a faster SM depletion in mutated, SPT-defective LY-B, than in myriocin-treated CHO cells, the latter containing a full amount of SL at the start of the experiment.

In parallel, PM patches of myriocin-treated and non-treated CHO cells were also visualized using lysenin-mCherry. PM patches of CHO cells treated with 2.5 µM myriocin and grown in deficient medium for 72 h (Fig. S2A) exhibited little lysenin-mCherry signal dots (indicated with white arrows), while the non-treated CHO cell PM patches grown in the same medium were extensively stained (Fig. S2B).

In summary, both non-treated CHO and LY-B cell lines grew to a similar extent in standard medium (Fig. 1A). Myriocin was effective in reducing cell growth rates only for CHO cells grown under limiting SL conditions. When using SL-deficient medium, myriocin-treated CHO cell growth was similar to that of LY-B and lower than that of non-treated CHO cells (Fig. 1A). Control growth of myriocin-treated cells in SL-deficient medium was recovered when the culture broth was supplemented with a proper SL (Fig. 1B). Lysenin fluorescence was largely decreased in myriocin-treated (but not in non-treated) CHO cells in the first 24 h of growth in deficient medium. Nevertheless, they contained higher SM amounts compared to non-treated LY-B cells (grown in deficient medium) and 72 h were needed to achieve a full lysenin-mCherry signal depletion equivalent to the one observed in LY-B cells (Figs. 2, 3, S2).

#### Myriocin effect on sphingosine-1-phosphate synthesis

Through abolition of SPT activity, myriocin could decrease sphingosine concentrations in the cell, and consequently lower the levels of sphingosine-1-phosphate. Since the latter is a well-known promoter of cell growth, changes in sphingosine-1-phosphate concentration could also influence the cell growth effects that we had attributed to SL deficiency in the growth media. To explore this possibility, the synthesis of sphingosine-1-phosphate by sphingosine kinase was assayed^13^ in the presence or absence of myriocin. The results show that, under our conditions, sphingosine kinase activity is not modified by myriocin (Fig. S3).

### Membrane lipid order decreases with sphingolipid restriction

Laurdan generalized polarization (GP) provides an estimation of membrane lipid molecular order^22^. To evaluate how SPT activity suppression affected the rigidity/fluidity of cell membranes, laurdan GP experiments were performed. GP values of myriocin-treated and non-treated CHO cells PM patches were measured using two-photon microscopy.

**Figure 4:**
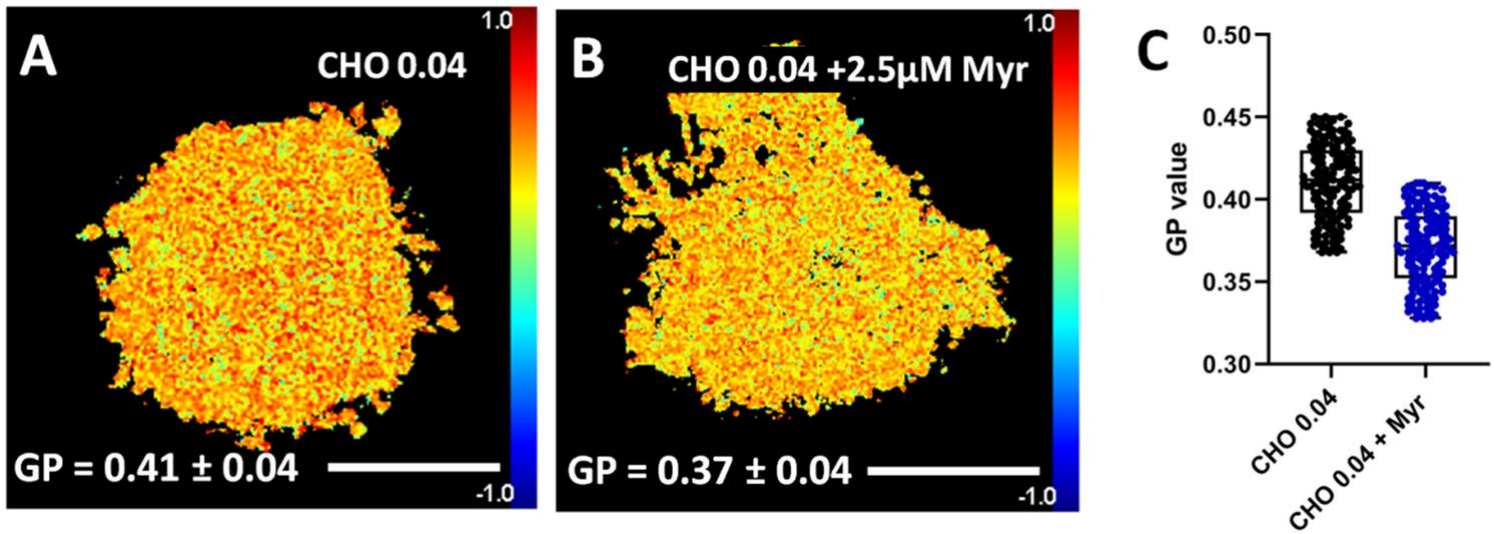
Two-photon microscopy images of PM patches stained with laurdan for GP measurements. PM patches of non-treated (**A**) and myriocin-treated (**B**) CHO cells grown in deficient medium. (**C**) Box plot graph of GP values of non-treated (black) and myriocin-treated (blue) CHO cell PM patches. GP are given as average values ± S.D. Three independent experiments were performed; n = 150 (50 PM patches × 3 replicas). Statistical significance (GP of control vs. myriocin-treated cells) was calculated with the Student’s t-test, *P* = 0.03. Bar = 30 µm.

Figure 4A depicts a PM patch from a CHO cell grown in deficient medium and stained with laurdan. In the − 1 to + 1 color scale at the right hand of the picture, the red color and a value close to + 1 indicated a more rigid/ordered region, while blue color and a value close to − 1 indicated a more fluid region. The GP value of PM patches derived from CHO cells treated with 2.5 µM myriocin and grown in deficient medium for 72 h (Fig. 4B) decreased on average from 0.41 to 0.37 compared with the non-treated CHO cells (Fig. 4A) grown in the same medium. Figure 4C shows a box plot graph of individual GP values of non-treated (black) and myriocin-treated (blue) CHO cell PM patches. The Student´s t test showed that a statistically significant difference existed between myriocin-treated and non-treated CHO-cell PM patches.

**Figure 5:**
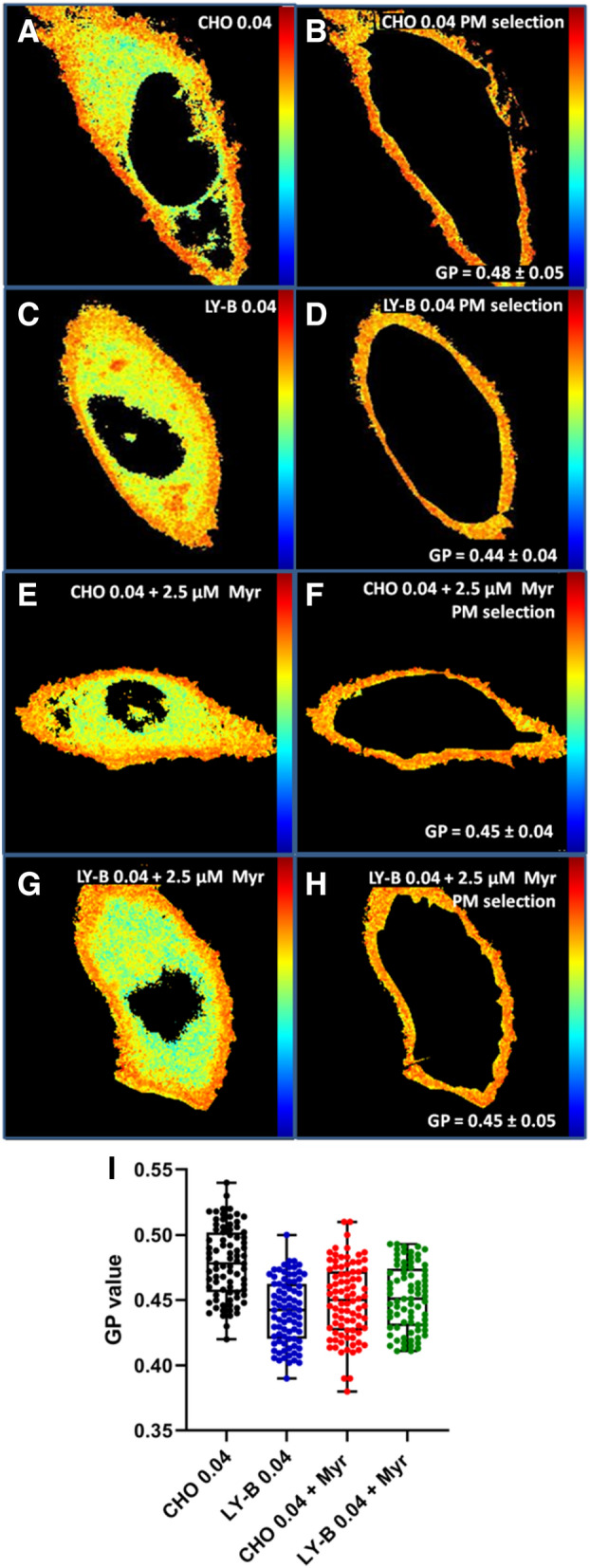
Laurdan staining and GP processing. A representative CHO cell grown in deficient medium (**A**), and the PM selection of the cell in panel A (**B**). A representative LY-B cell grown in deficient medium (**C**), and the PM selection of the cell in panel C (**D**). A representative 2.5 µM myriocin-treated CHO cell grown in deficient medium (**E**), and the PM selection of the cell in panel E (**F**). A representative 2.5 µM myriocin-treated LY-B cell grown in deficient medium (**G**), and the PM selection of the cell in panel E (**H**). Box plot graph of GP values of non-treated and myriocin-treated CHO (black and blue dots) and LY-B (red and green dots) PM selection values, including average values ± S.D. Three independent experiments were performed; n = 75 (25 PM selections × 3 replicas). Treated vs non-treated CHO cell *P* = 0.027 (**I**). Statistical significance was calculated with the Student´s t-test.

In Fig. 5 whole cells stained with laurdan and the corresponding optically selected PM pixels are shown. In whole cell images (Fig. 5A,C,E and G) two different regions could be distinguished, the more ordered PM and the more disordered/fluid intracellular membranes. In a previous work we showed that the two GP values were around 0.5 for PM and 0.2 for intracellular membranes^21^, indicating that the PM was less fluid than the intracellular membranes, perhaps due to the high Chol concentration in this membrane, in turn putatively related to its barrier role^23^.

PM pixels of treated and non-treated CHO and LY-B cells grown in SL-deficient medium for 72 h were measured for GP (Fig. 5B,D,F and H). The GP value of the PM region of myriocin-treated CHO cells decreased from 0.48 (Fig. 5B) down to 0.45 (Fig. 5F), while treated (Fig. 5H) and non-treated (Fig. 5D) LY-B cell PM GP value was constant at around 0.45. Figure 5I shows a box plot graph of GP values of non-treated and myriocin-treated CHO (black and blue dots) and LY-B (red and green dots) PM selection. Student´s t test revealed that the difference between myriocin-treated CHO cells grown in SL-deficient medium was significantly lower. On the contrary, there was no statistically significant difference between treated and non-treated LY-B cell PM.

Note that the average GP was clearly higher in the PM of whole cells (0.45–0.48) (Fig. 5) than in PM patches (0.37–0.41) (Fig. 4). It would be possible that the same membrane gave rise to a different GP value when the membrane was (roughly) spherical and when it was flat, lateral tension being zero in the latter case. In addition, cytoskeleton effects in the whole cells could be invoked as an explanation.

### Breakthrough force of myriocin-treated CHO cells plasma membranes is decreased

**Figure 6:**
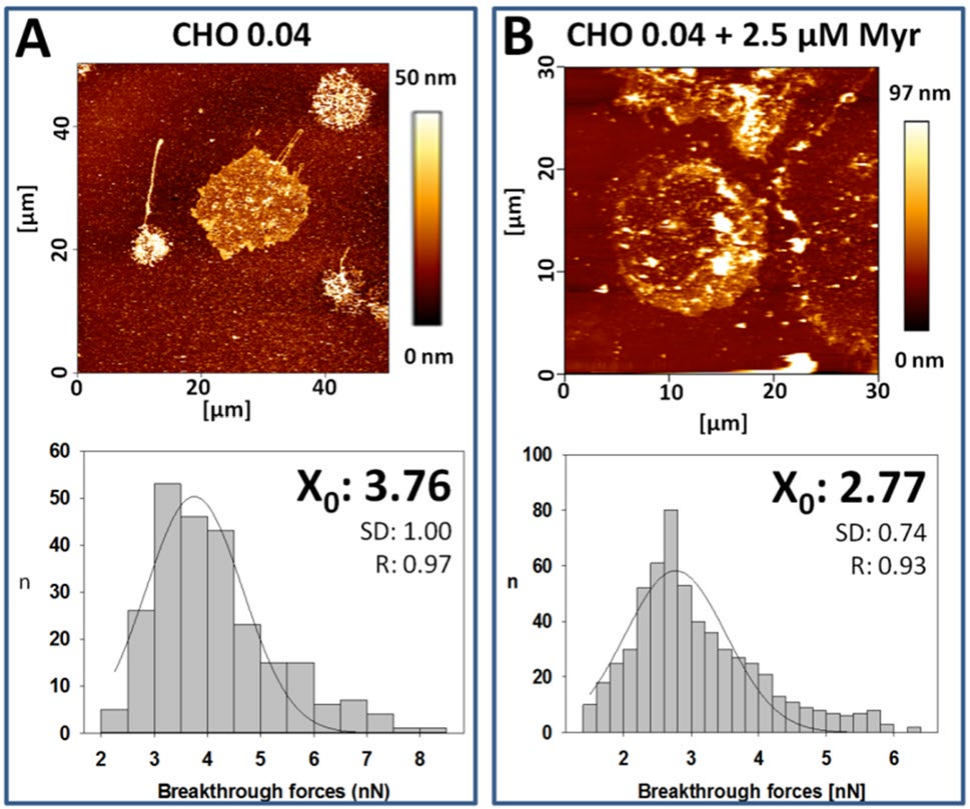
Topographic image and breakthrough force distributions of PM patches. PM patches from non-treated (**A**) and myriocin-treated (**B**) CHO cells grown in deficient medium. Three independent experiments were performed; n = 150–170 (50–60 breakthrough forces × 3 replicas. Statistical significance was calculated with the Student´s t-test, *P* = 0.0008.

Figure 6 shows the topology and breakthrough force distribution of PM patches derived from non-treated (Fig. 6A) and myriocin-treated (Fig. 6B) CHO cells grown in deficient medium for 72 h. According to topographic images, even if membrane components other than the bilayer, perhaps (glyco)proteins or protein aggregates, gave rise to protruding elements, a minimum thickness of 4–5 nm that corresponds to the thickness of a lipid bilayer was measured^24^. Other membrane components, perhaps (glyco)proteins or protein aggregates, might give rise to the thicker elements in the AFM pictures^13^. No statistically significant difference in thickness was observed between treated and non-treated CHO cells.

The breakthrough force distributions showed a significant difference between the PM patches derived from non-treated (3.76 nN; Fig. 6A) and treated (2.77 nN; Fig. 6B) CHO cells grown in deficient medium. This result is similar to the one obtained in Monasterio et al.^13^ when LY-B cell PM patches exhibited a large decrease, from 4.64 nN to 2.98 nN, when grown in normal or deficient medium^13^.

Breakthrough force measurements were in good agreement with laurdan GP values (Fig. 4). PM patches exhibited the expected behavior, the myriocin-treated CHO cells grown in deficient medium showing lower values in both parameters (Figs. 4 and 6), compared with non-treated cells.

### Lipidomic analysis of myriocin-treated vs. non-treated CHO cells

**Figure 7:**
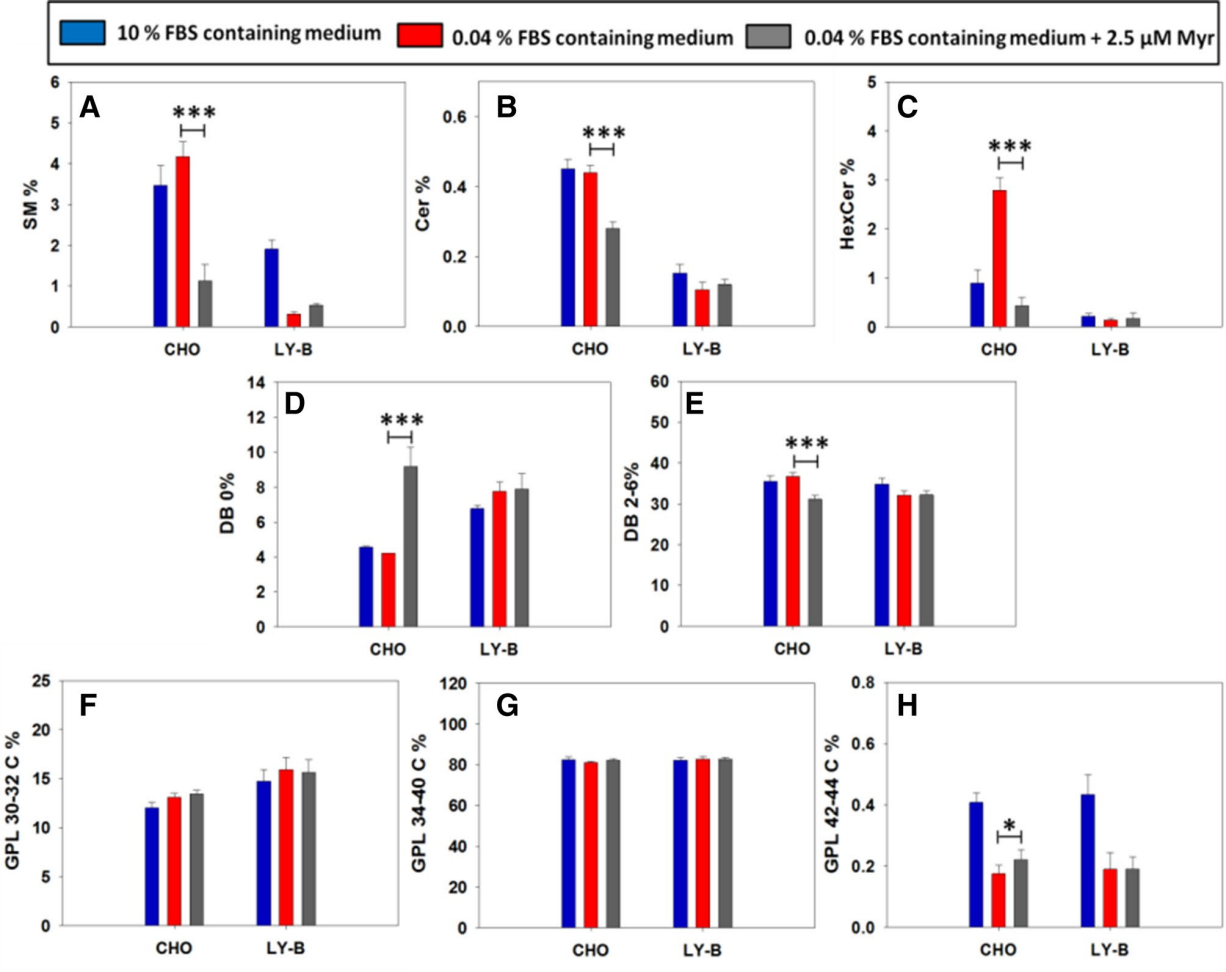
Myriocin treatment effects on the lipid composition of CHO ad LY-B PM patches. Total SM (**A**), Cer (**B**) and HexCer (**C**). Fully saturated (DB = double bond) (**D**) and polyunsaturated (**E**) GPL. Short-chain (30-32C) (**F**), long-chain (34-40C) (**G**) and very-long chain (42-44C) GPL (**H**). Only selected lipids are included in the figure, a comprehensive description of the various lipid compositions can be seen in the Supplementary Material Table S1. n = 3. Statistical significance (control vs. myriocin-treated) was calculated with the Student´s t-test: *P < 0.05; ***P < 0.001.

To understand the existing membrane order/disorder differences between myriocin-treated and non-treated CHO cells and their homeostatic regulation capacity, a lipidomic study of PM patches (Fig. 7) and whole cells (Fig. S4) was performed. A comprehensive description of the various lipid compositions can be seen in the supplementary material Table S1. The data are given in mole% of the lipid extract.

#### Plasma membrane patches

Some specific lipidomic results can be highlighted. Among the various SL classes, SM (by far the most abundant SL), Cer (an important SL in cell signaling), and HexCer (precursor of the complex glycosphingolipids) were selected (Fig. 7A–C). All three (SM, Fig. 7A; Cer, Fig. 7B and HexCer, Fig. 7C) significantly decreased in CHO cell PM patches (0.04% FBS) when treated with 2.5 µM myriocin (73% decrease for SM, 37% for Cer, and 85% for HexCer). However, the corresponding SL values of LY-B patches remained unchanged, or even increased slightly (Fig. 7A–C), perhaps because of their low values in non-treated cells: even if SM (Fig. 7A), Cer (Fig. 7B), and HexCer (Fig. 7C) amounts were largely decreased in myriocin-treated CHO cell PM patches, they were still twice (or larger) those of the non-treated LY-B cells (0.04% FBS). The latter observation is in agreement with the lysenin staining images in Figs. 2, 3, S2.

Glycerophospholipid (GPL) acyl chain saturation and length also play an important role in the membrane bilayer physical properties, specifically on its disorder/fluidity, unsaturated and shorter acyl-chain GPL increasing membrane fluidity^25^. In Fig. 7D,E the distribution of GPL saturation degree is shown. While in myriocin-treated LY-B cells GPL saturation degree in PM patches remained unchanged, in myriocin-treated CHO cells the fraction of fully saturated GPL increased (Fig. 7D) and that of polyunsaturated GPL (2–6 double bonds) decreased (Fig. 7E). In addition, PM patches from non-treated LY-B cells grown in deficient medium had a similar GPL saturation distribution than those from treated CHO cells grown in the same medium (Fig. 7D,E).

Considering GPL chain-length distribution, when myriocin-treated CHO cells were grown in deficient medium, their short 30–32 C acyl-chain GPL remained unchanged (Fig. 7F). Simultaneously, a slight but statistically significant increase in very long (42-44C) acyl-chain GPL was observed (Fig. 7H).

#### Whole cell extracts

The lipidomic distribution of treated and non-treated whole CHO and LY-B cells grown in deficient media is shown in Fig. S4. The results are remarkably similar to those found with the corresponding plasma membrane preparations, perhaps suggesting an active intracellular traffic.

#### Cholesterol

Cholesterol was determined by mass spectrometry separately from the fatty acid-containing lipids, as indicated under Methods. The results are given in Table 1. Decreasing FBS concentration in the growth medium from 10% to 0.04% decreased cholesterol concentrations in the cell membranes by about one-half. Since FBS is a major source of lipids and proteins for cell growth, the drastic reduction from 10% to 0.04% in the SL-deficient medium induces partial cell starvation, and Chol synthesis is known to decrease under fasting conditions^26–28^. However, as cholesterol synthesis was already limited because of the starvation conditions, myriocin failed to significantly affect the amount of cholesterol in cells grown under limiting SL concentrations.

**Table 1:** Myriocin effect on cholesterol concentration in whole cell or plasma membrane lipid extracts from CHO or LY-B cells grown under limiting SL concentration.

Cell type	[FBS]	Myriocin	Mole % Whole cells	Mole % PM patches
CHO	10%	–	3.12 ± 0.17*	5.91 ± 0.29*
CHO	0.04%	–	1.56 ± 0.19	3.12 ± 0.20
CHO	0.04%	2.5 µM	1.36 ± 0.07	3.43 ± 0.42
LY-B	10%	–	3.05 ± 0.13*	8.65 ± 0.90*
LY-B	0.04%	–	1.22 ± 0.01	3.85 ± 0.51
LY-B	0.04%	2.5 µM	1.26 ± 0.03	3.65 ± 0.10

Results are given as mole percent of total lipids in sample. Average ± S.D. (n = 3).

*Results taken from Monasterio et al*.*^13^.

**Figure 8:**
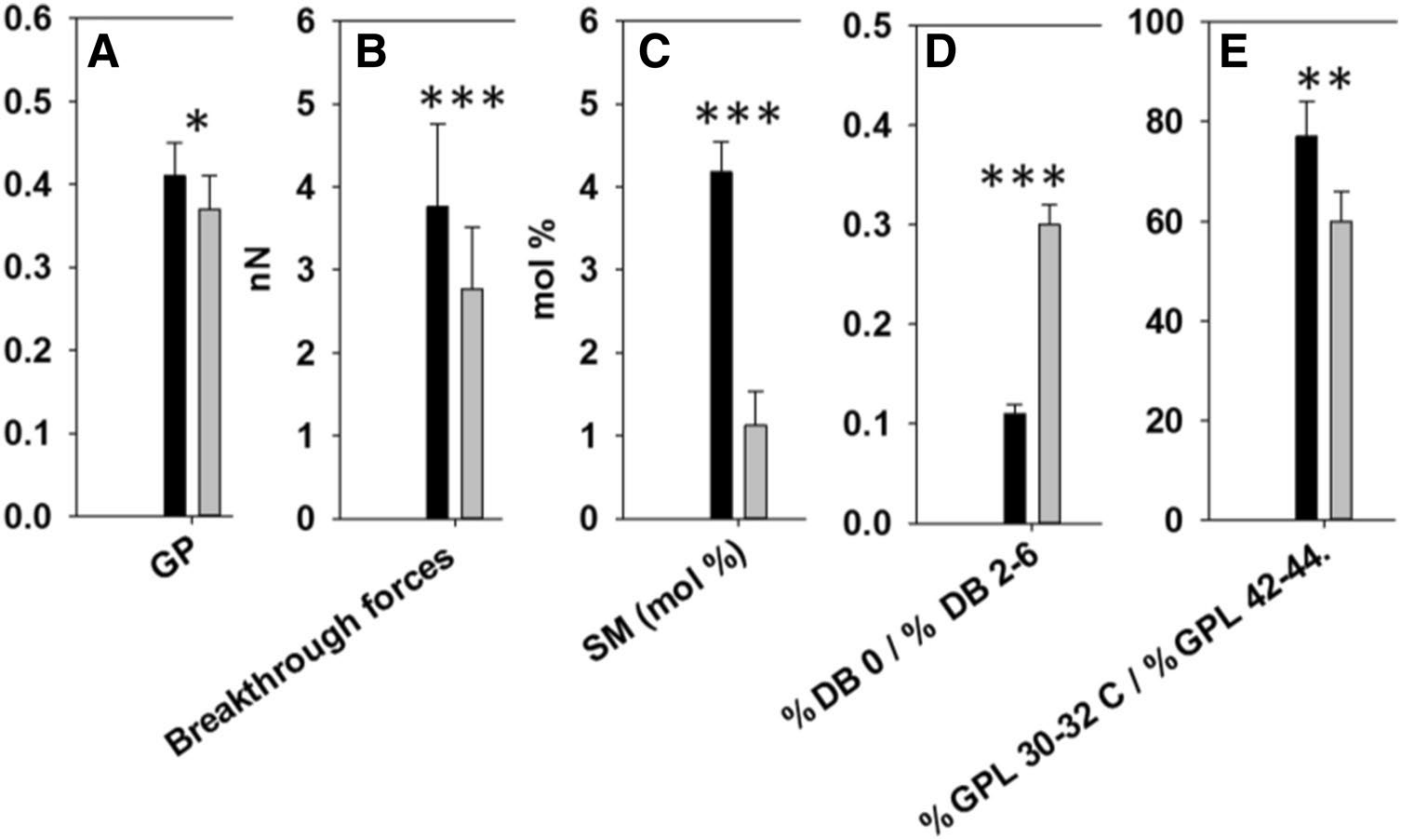
A correlative presentation of myriocin effects on the plasma membranes of CHO cells grown on SL-deficient medium. Black bars: control, non-myriocin treated cells; grey bars: myriocin-treated cells. (**A**) Laurdan general polarization in PM patches (data from Fig. 4). (**B**) Bilayer breakthrough forces obtained by AFM in the force-spectroscopy mode (data from Fig. 6). (**C**) Mole percent SM contents in PM patches (data from Table S1 and Fig. 7A). (**D**) Saturated/unsaturated fatty-acyl mole ratio (data from Table S1 and Fig. 7D, E). (**E**) Short-chain/very-long-chain fatty-acyl mole ratio (data from Table S1 and Fig. 7 F, H). Significance (control vs. myriocin-treated cells) according to Student’s t-test: *P < 0.05; **P < 0.01, ***P < 0.001.

*In summary,* myriocin effects on lipid composition were almost exclusively detected under conditions when myriocin hampered cell growth, i.e. CHO cells grown under limiting SL conditions. SM (Fig. 7A), Cer (Fig. 7B), and HexCer (Fig. 7C) concentrations in the PM were markedly lower in myriocin-treated than in non-treated CHO cells. Even if treated cells exhibited a large SL decrease, their levels were still larger than those of non-treated LY-B cell PM patches. According to GPL acyl chain length and saturation, myriocin-treated CHO cells synthesized longer (Fig. 7H) and more saturated (Fig. 7D) GPL, perhaps a homeostatic response to myriocin-mediated SM depletion. The correlation between CHO cell PM changes in lipid composition and in biophysical properties is graphically expressed in Fig. 8.

## Discussion

The main aim of this paper was to compare two different approaches to suppress sphingolipid synthesis in mammalian cells, namely genetic suppression of SPT gene functionality and chemical inhibition of the said enzyme. The underlying idea was that each of those procedures was potentially able to modify cell metabolism in ways different from originally intended, e.g. through hitherto undescribed effects on other genes, or through direct interaction with other intermediary metabolites. The comparative study of both experimental approaches could reveal differences attributable to side effects, or else it could confirm that both techniques led to the specific inhibition of SPT, in the absence of detectable further effects.

### Myriocin effects on cell growth and composition

Most natural compounds with potent SPT-inhibitory properties, such as myriocin, are structural analogues of sphingosine^29^, a bioactive molecule modulating a variety of cell functions^30,31^. The polyfunctionality of sphingosine might suggest that myriocin would have additional biological activities apart from the inhibitory effect of the de novo synthesis of SL. When an inhibitory compound is highly specific, the cellular effect that this compound produces should be the same as the one caused by the genetic inactivation of the target, the SPT enzyme in our case. On the contrary, if the cellular response to a drug is different from the one achieved by genetic inactivation of the target enzyme, the drug might have additional effects. In a previous investigation, we used a genetically modified CHO cell line defective in SPT (the LY-B cell line) to perform biophysical measurements and check their homeostatic response to SL deprivation^13^. In the current study, we have used myriocin to specifically inhibit SPT and compare the chemical inhibition results with the ones obtained after genetic modification (LY-B results)^13^, thus addressing myriocin specificity against the SPT enzyme activity.

When cell growth of myriocin-treated CHO and non-treated LY-B cells were compared, only slight differences were found. Myriocin-treated CHO cells were able to recover to a large extent the control (non-treated CHO cell) growth rates when the SL-deficient medium was supplemented with sphinganine (Figs. 1B and S1F). When externally supplied to the culture medium, sphinganine is utilized for synthesis of Cer and complex SL in CHO cells, thereby bypassing the SPT reaction inhibition. A similar behavior was observed with SM supplementation (Fig. S1C). Nevertheless, control (non-treated CHO grown in deficient medium) values were not reached (Fig. 1B). This is in agreement with other studies, in which growth rate recovery was not achieved either when the SPT inhibitor was L-Cycloserine or β-chloro-L-alanine^32^ instead of myriocin. SPT inhibition could lead to the decrease of sphingosine-1-phosphate cell levels. Sphingosine-1-phosphate has been described as a second messenger favoring cell proliferation and survival^33–35^. However, in our hands, myriocin did not modify sphingosine-1-phosphate levels (Fig. S3), thus it is unlikely that the latter metabolite was influencing the overall myriocin effects described in Figs. 1 and S1.

Alternatively, the different growth rates of myriocin-treated and non-treated CHO cells (Fig. 1A) may be related to the over-production of HexCer observed in non-treated CHO, but not in myriocin-treated CHO cell patches upon lowering FBS concentration in the medium (Fig. 7C). HexCer is at the origin of the complex glycosphingolipid biosynthetic pathway^36^. Glycosphingolipids are essential cell components involved, among other effects, in cell growth and differentiation^37^. However, in general, the external addition of sphingosine is not expected to mimic completely the de novo synthesis: the “initial” location of sphingosine is different and the intracellular traffic and distribution are also expected to be different as well as the biophysical changes at the PM and in the membrane of different organelles, probably affecting cellular signaling and homeostasis in different ways. These more general effects should also be considered.

Comparing the SL levels in PM patches derived from myriocin-treated and non-treated CHO cells grown in deficient medium, it can be concluded that the former exhibit significantly lower SL values. Three representative SL, namely SM (Fig. 7A), Cer (Fig. 7B), and HexCer (Fig. 7C), were largely decreased, by 73%, 37%, and 85% respectively. These results are in good agreement with the ones published by Ziulkoski et al*.*^38^, who achieved a 40%-61% SM decrease using fumonisin B1 and β-chloroalanine. An important myriocin effect was that a more rapid SM depletion was obtained in mutated defective- SPT-containing LY-B than in CHO cells (Figs. 2, 3, S2). In addition, SM, Cer and HexCer levels, even if their amounts were markedly decreased by myriocin treatment, were still larger in treated CHO cells than in non-treated LY-B cells (0.04% FBS).

### Lipid composition and physical properties

SL depletion is probably what makes PM patches derived from myriocin-treated CHO cells to show a decreased laurdan GP (indicating a decreased lipid order, or increased bilayer fluidity) (Figs. 4 and 5) and a lowered breakthrough force distribution (increased membrane penetrability) (Fig. 6). GP images of PM patches derived from myriocin-treated CHO cells appeared to be homogenous, but the presence of nanodomains could not be ruled out because of the spatial and temporal resolution limit of conventional two-photon microscopy^39–41^_._

In addition, a comparison of myriocin-treated and non-treated CHO cells showed changes in their GPL distribution. Myriocin-treated cells contained more fully saturated (Fig. 7D) and less polyunsaturated (Fig. 7E) GPL than non-treated ones. This could partially help to maintain membrane rigidity under conditions of low SM. This observation is in accordance with results obtained with the LY-B cells containing a mutated, non-functional SPT^13^. According to chain length distribution, LY-B cells grown in deficient medium contain more short-chain fatty acids (C30-32C per GPL molecule) than myriocin-treated CHO cells^13^. A summary of the chemical and physical changes induced by myriocin is shown in Fig. 8.

### Chemical vs. genetic suppression of SPT activity

This study presents a phenotypic comparison between mutant cells (LY-B) specifically defective in a cellular function (de novo SL synthesis), and wild-type CHO cells, exposed to a chemical inhibitor of the SPT activity function (myriocin) as a rational approach for evaluating how specific an inhibitor compound can be. The main difference between the two approaches is that LY-B cells lack a functional SPT complex because they cannot synthesize the LCB1 subunit, while the myriocin-mediated inhibition only blocks the SPT function, but the protein is still present. This may have important implications, for example the drug-inhibited protein may lack a certain activity but may still interact with some binding partners, as many proteins have multiple activities and/or functions and may participate in multiple disparate pathways, processes, and/or multiprotein complexes and structures^42–45^.

Considering the above data, very similar results were achieved with both the pharmacological and genetic tools. Myriocin-treated SM-depleted CHO cells reacted to maintain membrane order undergoing a homeostatic response, although they achieved it only partially, as their PM were more fragile when grown in SL-deficient medium. The same occurred with non-treated LYB cells^13^.

The capacity shown by certain cells, e.g. myriocin-treated CHO cells or genetically modified LY-B cells, to grow under extremely demanding low concentrations of SL opens the way to a variety of functional studies on the role of SL in membranes. Nevertheless, natural compounds as myriocin or fumonisin may display toxicity in humans^47^, hence the need for chemical inhibitors more suitable for human studies. A therapy acting on SPT activity might show good results in the treatment of cardiovascular diseases, such as atherosclerosis^46,47^. Other studies provided evidence that inhibition of de novo Cer synthesis improves glucose homeostasis and enhances whole-body insulin responsiveness in rodent models of type-2 diabetes^48^. Moreover, it has been shown that it reduces body weight, ameliorates glucose homeostasis, and reverses hepatic steatosis in diet-induced nonalcoholic fatty liver disease^49^. In addition, it has been seen that the blockage of de novo synthesis of Cer and HexCer significantly suppresses neurodegenerative phenotypes associated with α-synuclein overexpression^50^.

In conclusion, the myriocin inhibition chemical method and the genetic deletion method, as applied to SPT, lead to very similar effects on cell growth, plasma membrane lipid composition and membrane biophysical properties. This speaks in favor of both approaches being highly specific and virtually free of side effects when applied to the preparation of cells in which de novo sphingolipid synthesis had been suppressed. In turn, such cells could be of use in the study of important and frequent human pathologies.

## Methods

### Materials

Wild type CHO (ATCC, Manassas, Virginia, U.S.) and a serine-SPT deficient CHO cell line, known as LY-B^8^ (RIKEN BioResource Research Center, Koyadai, Japan), were used. Cell culture products; DMEM:F12 (Dulbecco's Modified Eagle *Medium*: Nutrient Mixture *F-12)* FBS (Fetal Bovine Serum), penicillin, streptomycin, and GlutaMax suplement were purchased from Thermofisher (Waltham, MA). Organic solvents were from Thermofisher (Waltham, MA). All fluorophores; were purchased from Thermofisher (Waltham, MA). Salts for buffer preparation (KCl, NaCl, CaCl_2,_ HEPES), myriocin, L-α-phosphatidylcholine from chicken egg (PC), sphingomyelin from chicken egg (SM) and D-erythro-sphinganine (sphinganine) were purchased from Sigma-Aldrich (St. Louis, MO, U. S.). All other reagents (salts and organic solvents) were of analytical grade.

### Cell growth

CHO and LY-B^8^ cells were grown on DMEM:F12 (Dulbecco's Modified Eagle Medium: Nutrient Mixture F-12*)* medium containing 10% FBS (Fetal Bovine Serum), 100 U/ml penicillin, 100 U/ml streptomycin, and 6 mM glutamine (GlutaMax supplemented) at 37 °C and 5% CO_2_ humidified atmosphere. All cell culture products were purchased from Thermofisher (Waltham, MA, US).

#### Myriocin treatment

CHO and LY-B cells were first seeded in DMEM:F12 medium containing 10% FBS, 100 U/ml penicillin and 100 U/ml streptomycin, and 6 mM glutamine (this medium will be referred as ‘standard medium’). After 24-h the standard medium was changed by DMEM:F12 medium containing either 10% or 0.04% FBS, 100 U/ml penicillin and 100 U/ml streptomycin and 6 mM Glutamine (the medium containing 0.04% FBS will be named ‘FBS-deficient’ or ‘SL-deficient medium’). Then myriocin (SigmaAldrich, St.Louis, MO, US) dissolved in DMSO was added to a final concentration of 2.5 µM^51^ and cells were cultured for 24, 48 or 72 h before any experiment was performed.

### Growth rate and viability tests

#### Cell growth

2.65 × 10^5^ cells were seeded in 25 cm^2^ flasks in standard medium and grown for 24 h until 15–25% confluence. Then, the standard medium was discarded, cells were washed twice with PBS, and the appropriate medium (standard or deficient, with or without 2.5 µM myriocin)^51^ was added. Cells were grown for 24, 48 or 72 h. Quantification was performed by cell counting with a hemocytometer (BioRad TC20 Automated Cell Counter, Hercules, CA).

#### Viability test

Flow citometry experiment was performed to evaluate how the myriocin treatment affected cell viability^52^. Cells were stained with Annexin-V-FITC and propidium iodide as indicated in the manual of the annexin V-FITC detection kit (CalbioChem, Darmstadt, Germany) and fluorescence was measured using a FACS Calibur flow cytometer (Becton–Dickinson, Franklin Lakes, NJ) as in Ahyayauch et al*.*^53^. Annexin V-FITC fluorescence intensity was measured in fluorescence channel FL-1 with λ_ex_ = 488 nm and λ_em_ = 530 nm, while FL-3 was used for propidium iodide detection, with λ_ex_ = 532 nm and λ_em_ = 561 nm. All measurements were performed in triplicate. Data analysis was performed using Flowing Software, Turku Centre for Biotechnology, Unversity of Turku, Finland, version: 2.5.1, http://flowingsoftware.btk.fi/.

### Sample preparation

Intact cells (whole cells) and PM patches have been used. Intact cells were grown as explained above. PM patches were isolated by a modification^21^ of the protocol described by Bezrukov et al*.*^20^. In summary, cells were seeded at approximately 50% confluence and incubated for 2 h so that they adhered to the support. After incubation, two washing steps were performed using cold TBS (Tris Buffer Saline: 150 mM NaCl, 25 mM Tris–HCl, 2 mM KCl) to discard non-attached cells. Then, cold distilled water was added for 2 min to induce cell swelling. Mechanical cell disruption was achieved using a pressure stream from a 20-ml syringe coupled to a 19X1-1/2(TW)A needle. In the process, intracellular content was released, while PM stayed attached to the support. Several washing steps were performed to discard the released intracellular content. Purification quality was checked using Di-4-ANEPPDHQ (λ_ex_ = 465 nm, λ_em_ = 635 nm) as a general fluorescent staining, together with organelle-specific fluorophores as described in Monasterio et al*.*^21^*.* Nuclei were stained using 2.8 µM Hoechst 33,342 (λ_ex_ = 361 nm, λ_em_ = 497 nm) for 10 min at 37 °C, Golgi apparatus was stained using 10 µM BODYPY FL C5-ceramide (λ_ex_ = 500 nm, λ_em_ = 510 nm) for 30 min at 37 °C, and mitochondrial staining was performed with 0.75 µM Mitotracker Green (λ_ex_ = 488 nm, λ_em_ = 510 nm) for 30 min at 37 °C. All fluorophores were purchased from Thermofisher (Waltham, MA). Images were taken with a Leica TCS SP5 II microscope (Leica Microsystems GmbH, Wetzlar, Germany) at room temperature with ImageJ software. The fluorescence intensities of the various markers were comparatively measured in PM patches and intact cells, so that specific organelle contamination could be estimated.

### SM quantification with lysenin

#### Lysenin-mCherry expression and purification

The non-toxic monomeric C-terminal domain of the SM-specific toxin, non-toxic- (NT) lysenin, was expressed and purified as described by Carquin et al*.*^54^. Briefly, the expression plasmid pET28/lysenin encoded NT-lysenin as a fusion protein with an N-terminal 6xHis-tag followed by the monomeric red fluorescent protein mCherry. The plasmid was expanded in *Escherichia coli* BL21 (DE3) and the recombinant protein was expressed in lysogeny broth (LB) medium at 16ºC for 72 h in the presence of 0.4 mM isopropyl β-D-thiogalactoside. Bacterial extracts were prepared as described^55^ and the recombinant protein was purified using an Ni–NTA Superflow cartridge (Qiagen, Hilden, Germany) and eluted with imidazol^56^. Fraction analysis by SDS-PAGE revealed recombinant NT-lysenin with the expected size (45 kDa). The most enriched fractions were pooled, concentrated, and desalted. The aliquots were stored in 20 mM NaCl and 25 mM Hepes pH 7.2 and 5% glycerol at − 80 °C. Protein concentration was calculated by measuring absorbance at 280 nm.

#### SM staining and quantification with lysenin-mCherry

SM in whole cells and PM patches was stained with lysenin-mCherry and samples were visualized using a confocal microscopy Nikon D-ECLIPSE C1 (Nikon, Melville, NY). Samples were stained with lysenin-mCherry at 100 µM prior to visualization. PM patches, but not whole cells, were first stained with 100 µM NBD-PE as a control for all-lipid staining. A washing step was performed with PBS, and lysenin-mCherry was added at 100 µM final concentration. Whole-cell mCherry signal was also quantified using a FL-3 FACS Calibur flow cytometer (Becton–Dickinson, Franklin Lakes, NJ) with λ_ex_ = 532 nm and λ_em_ = 561 nm.

### Laurdan General Polarization (GP)

Laurdan is a fluorescence polarity probe whose emission undergoes a spectral shift due to the reorientation of water molecules in the glycerol backbone region of the membrane, and this shift can be correlated to the lipid phase^22^. In the gel phase, when little water is present, laurdan maximum emission is around 440 nm, whereas in the liquid crystalline phase the spectrum is red shifted to around 490 nm. Intact cells and PM patches have been used to compare the laurdan fluorescence of myriocin treated or non-treated CHO and LY-B cells. Samples were stained with 5 µM laurdan (Molecular Probes, Eugene, OR) for 5 min and two PBS washing steps were performed prior to cell visualization.

#### Image acquisition and analysis

Images were acquired and analysed as described in Monasterio et al*.*^13^. In summary, a Leica TCS SP5 II microscope (Leica Microsystems GmbH, Wetzlar, Germany) with a 63 × water-immersion objective (numerical aperture NA = 1.2) was used and samples were imaged at 512 × 512 pixel and 400 Hz per scanning line. Equatorial planes were imaged to avoid photoselection effects. A pulsed titanium-sapphire (Mai-Tai Deepsee, Spectra-Physics) laser tuned at 780 nm was used for two-photon imaging of laurdan-labeled samples. Fluorescence emission was collected by non-descanned (NDD) hybrid detectors, as they offer higher sensitivity compared to descanned photomultipliers. The blue edge of the emission spectrum was collected by NDD 1 at 435 ± 20 nm and the red edge by NDD 2 at 500 ± 10 nm. Irradiance at the sample plane was ≈500 GW·cm^–2^ for two-photon excitation^57^. Three independent experiments were performed, taking 50 PM patch images in each of them. The software was Leica Application Suite Advances Fluorescence, Leica Microsystems CMS (Wetzlar, Germany), version: 2.6.3.8173, https://www.leica-microsystems.com/products/microscope-software/p/leica-application-suite/.

GP value of samples was calculated using GenPol, a home-made MATLAB (MathWorks, Natick, MA) -based software, version Unidad de Biofísica 18Dec15 https://www.mathworks.com/products/matlab.html. This software is available free from the authors, by request. Images were smoothed in each channel with 2 pixel averaging, and the GP value was calculated using the following Eq. ^58^:

$$ {\text{GP}} = \frac{{{\text{I}}_{{\text{B}}} - {\text{ G}}\cdot{\text{I}}_{{\text{R}}} }}{{{\text{I}}_{{\text{B}}} + {\text{G}}\cdot{\text{I}}_{{\text{R}}} }} $$

where I_B_ is the intensity collected by NDD 1, I_R_ is the intensity collected by NDD 2, and G is the correction factor. The G factor is calculated measuring the GP value of the same fluorophore concentration used in sample staining, dissolved in this case in pure DMSO^23^. The region of interest, i.e. the PM, was selected.

### Atomic force microscopy

Topographic images and force spectroscopy analysis of PM patches were performed. PM patches were prepared as previously described^20,21^, using this time polylysine-coated mica slips instead of glass-bottom dishes. PM patches were first stained using Di-4-ANEPPQHD to allow detection on the mica slip.

Samples were measured as described in Monasterio et al*.*^13^. In summary, contact mode AFM imaging has been used to study bilayer topography, looking at possible lateral segregation effects through bilayer thickness analysis. A NanoWizard II AFM (JPK Instruments, Berlin, Germany) was used to perform topographic measurements under contact mode scanning (constant vertical deflection). For measurements, the AFM was coupled to a Leica microscope and mounted onto a Halcyonics Micro 40 antivibration table (Halcyonics, Inc., Menlo Park, CA) and inside an acoustic enclosure (JPK Instruments, Berlin, Germany)^13,59^. V-shaped MLCT Si_3_N_4_ cantilevers (Bruker, Billerica, MA) with nominal spring constants of 0.1 or 0.5 N/m. The sample thickness was estimated by cross-section height analysis^60^.

For Force Spectroscopy, V-shaped MLCT Si_3_N_4_ cantilevers (Bruker, Billerica, MA) with nominal spring constants of 0.1 or 0.5 N/m were individually calibrated in a lipid-free mica substrate in assay buffer using the thermal noise method. After proper bilayer area localization by means of AFM topography and direct epifluorescence microscopy, force spectroscopy was performed at a speed of 1 μm/s. Force steps were determined for each of the indentation curves as reproducible jumps within the extended traces. A single event was observed in most cases. When a double event was detected, which we attribute to folded membrane areas, the sample was discarded. At least three independent sample preparations were scanned for each case and 50–100 curves were measured in each sample. Software for AFM was JPK Data processing, Bruker Corporation, version spm-5.0.131, www.jpk.com.

### Mass spectrometry analysis

Mass spectrometry analysis was performed essentially as described in Monasterio et al.^21^. A methodological summary follows.

#### Sample treatment

Lipid extraction was performed using a modified methyl *tert*-butyl ether (MTBE) protocol^61^. Briefly, cells or PM patches were washed with cold PBS and scraped off in 500 μl cold PBS on ice. The suspensions were transferred to a 2 ml tube and spun down at 3200 rpm for 5 min at 4 °C. After removing the PBS, samples were stored at -20 °C or directly used for further extraction. Then, 360 μl methanol was added and vortexed. A mixture of lipid standards (see Table 2) was added and samples were vortexed for 10 min at 4ºC using a Cell Disruptor Genie (Scientific Industries, Inc., Bohemia, NY). MTBE (1.2 ml) was then added and the samples were incubated for 1 h at room temperature with shaking (750 rpm). Phase separation was induced by adding 200 μl H_2_O. After 10 min incubation at room temperature, the samples were centrifuged at 1,000 × g for 10 min. The upper (organic) phase was transferred to a 13-mm screw-cap glass tube and the lower phase was extracted with 400 μl artificial upper phase (MTBE/methanol/water (10:3:1.5, v/v/v)). The two upper phases were combined and the total lipid extract was divided in 3 equal aliquots (one for phospholipids (TL), one for sterols (S) in 2-ml amber vials, and one for SL detection in a 13-mm glass tube) and dried in a Centrivap at 50 °C or under a nitrogen flow. The SL aliquot was deacylated by methylamine treatment (Clarke method) to remove glycerophospholipids. 0.5 ml monomethylamine reagent [MeOH/H_2_O/n-butanol/methylamine solution (4:3:1:5 v/v)] was added to the dried lipid, followed by sonication (5 min). Samples were then mixed and incubated for 1 h at 53 °C and dried (as above). The monomethylamine-treated lipids were desalted by n-butanol extraction. 300 μl H_2_O-saturated n-butanol was added to the dried lipids. The sample was vortexed, sonicated for 5 min and 150 μl MS-grade water was added. The mixture was vortexed thoroughly and centrifuged at 3200 × g for 10 min. The upper phase was transferred to a 2-ml amber vial. The lower phase was extracted twice more with 300 μl H_2_O-saturated n-butanol and the upper phases were combined and dried (as above).

**Table 2:** MS detection conditions for the different lipid classes.

Lipid class	Standard	Polarity	Mode	m/z ion	Collision energy
Phosphatidylcholine [M + H]^+^	DLPC	+	Product ion	184.07	30
Phosphatidylethanolamine [M + H]^+^	PE31:1	+	Neutral ion loss	141.02	20
Phosphatidylinositol [M − H]^−^	PI31:1	−	Product ion	241.01	44
Phosphatidylserine [M − H]^−^	PS31:1	−	Neutral ion loss	87.03	23
Cardiolipin [M − 2H]^2−^	CL56:0	−	Product ion	acyl chain	32
Ceramide [M + H]^+^	C17Cer	+	Product ion	264.34	25
Dihydroceramide [M + H]^+^	C17Cer	+	Product ion	266.40	25
Hexosylceramide [M + H]^+^	C8GC	+	Product ion	264.34	30
Hexosyldihydroceramide [M + H]^+^	C8GC	+	Product ion	266.40	30
Sphingomyelin [M + H]^+^	C12SM	+	Product ion	184.07	26

#### Glycerophospholipid and sphingolipid detection on a Triple Quadrupole Mass Spectrometer

TL and SL aliquots were resuspended in 250 μl chloroform/methanol (1:1 v/v) (LC–MS/HPLC grade) and sonicated for 5 min. The samples were pipetted in a 96-well plate (final volume = 100 μl). The TL were diluted 1:4 in negative-mode solvent (chloroform/methanol (1:2) + 5 mM ammonium acetate) and 1:10 in positive-mode solvent (chloroform/methanol/water (2:7:1 v/v) + 5 mM ammonium acetate). The SL were diluted 1:10 in positive-mode solvent and infused onto the mass spectrometer. Tandem mass spectrometry for the identification and quantification of SL molecular species was performed using Multiple Reaction Monitoring (MRM) with a TSQ Vantage Triple Stage Quadrupole Mass Spectrometer (Thermofisher Scientific, Waltham, MA) equipped with a robotic nanoflow ion source, Nanomate HD (Advion Biosciences, Ithaca, NY). The collision energy was optimized for each lipid class. The detection conditions for each lipid class are listed below (Table 2). Cer species were also quantified with a loss of water in the first quadrupole. Each biological replica was read in 2 technical replicas (TR). Each TR comprised 3 measurements for each transition. Lipid concentrations were calculated relative to the relevant internal standards and then normalized to the total lipid content of each lipid extract (mol %).

### Gas chromatography–mass spectrometry for cholesterol assay

Lipid extracts were analyzed by GC–MS as described previously^62^. Briefly, samples were injected into a VARIAN CP-3800 gas chromatograph equipped with a FactorFour Capillary Column VF-5 ms 15 m × 0.32 mm i.d. DF = 0.10, and analyzed in a Varian 320 MS triple quadrupole with electron energy set to –70 eV at 250 °C. Samples were applied to the column oven at 45 °C, held for 4 min, then raised to 195 °C (20 °C/min). Sterols were eluted with a linear gradient from 195 to 230 °C (4 °C/min), followed by rising to 320 °C (10 °C/min). Cholesterol was identified by its retention time (compared with an ergosterol standard) and fragmentation patterns, which were compared with the NIST library.

### Sphingosine kinase assay

Sphingosine kinase was assayed on CHO cells according to Lima et al.^63^, with NBD-sphingosine derivatives. The procedure is based on the different fluorescent properties of NBD-sphingosine (λ_ex_ = 474 nm, λ_em_ = 539 nm) and NBD-sphingosine-1-phosphate (λ_ex_ = 550 nm, λ_em_ = 584 nm). Briefly, on day 1 CHO cells are grown in standard medium, containing 10% FBS. On day 2 the medium is replaced with SL-poor medium (0.04% FBS) to cause a cellular decrease in sphingosine (among other SL). On day 3, 13 µM NBD-sphingosine is added, together with 2.5 µM myriocin when required. Cells are collected 1 h and 24 h after NBD-sphingosine addition, treated with lysis buffer (including protease inhibitor cocktail) and fluorescence emission spectra are recorded.

## Supplementary Information

Supplementary Information 1.

Supplementary Information 2.
