# Supplementary material for: Plasma membrane effects of sphingolipid-synthesis inhibition by myriocin in CHO cells: a biophysical and lipidomic study
Source: Sci Rep. 2022 Jan 19;12:955. doi: 10.1038/s41598-021-04648-z (PMC8770663; doi:10.1038/s41598-021-04648-z)
Supplement: Supplementary file 2 — Supplementary Information 2. [file 41598_2021_4648_MOESM2_ESM.pdf]

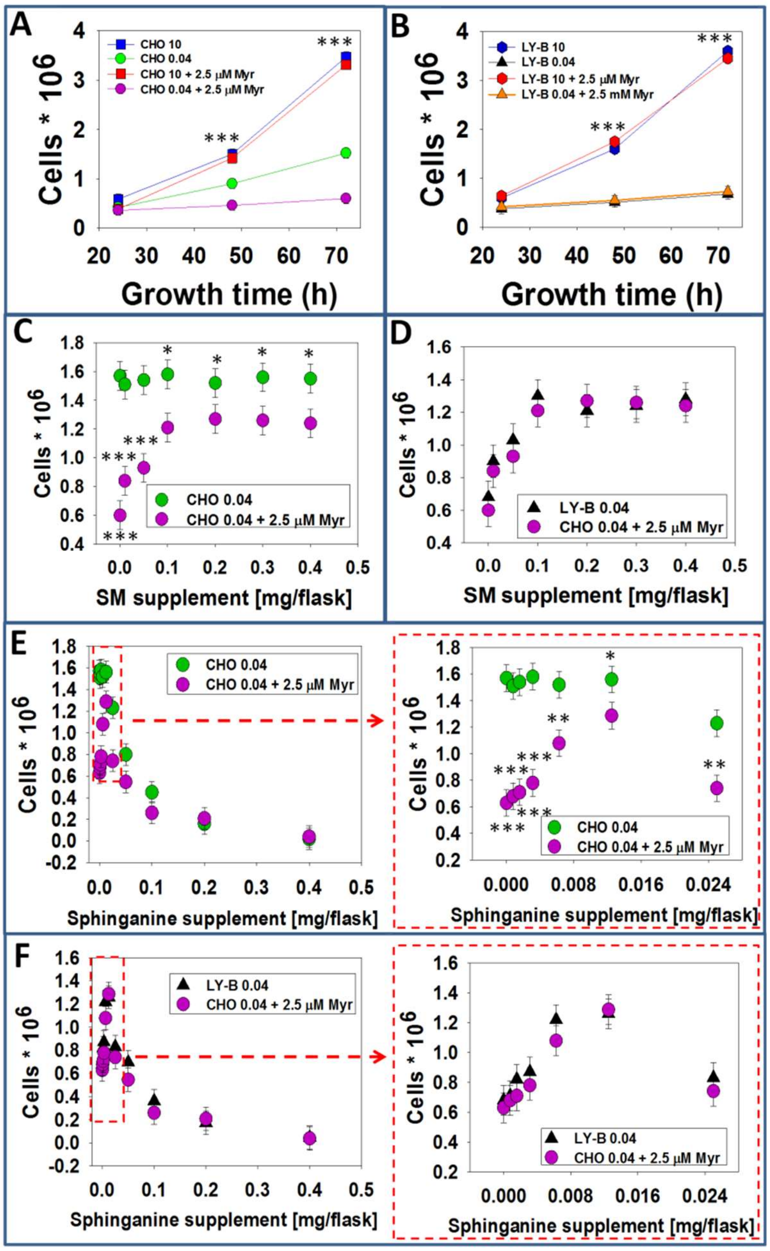

Figure S1. Growth of myriocin-treated and non-treated CHO and LY-B cells. CHO (**A**) or LY-B (**B**) cell growth as a function of time in 0 or 2.5 µM myriocin-containing, standard (10% FBS) or sphingolipid-deficient (0.04% FBS) medium (seeded cells: 0.25 * 10^6). Statistical symbols correspond to differences between purple circles (CHO 0.04 + 2.5µM Myr) and the other three samples in panel A and between triangles (LY-B 0.04 + 0 or 2.5 µM Myr) and circles (LY-B 10 + 0 or 2.5 µM Myr. (**C**) Myriocin-treated vs. non-treated CHO cell growth after 72 h in sphingolipid-deficient medium supplemented with SM (seeded cells: 0.25 * 10^6). (**D**) Myriocin-treated CHO vs. non-treated LY-B cells growth after 72 h in sphingolipid-deficient medium supplemented with SM (seeded cells: 0.25 * 10^6). Myriocin-treated vs. non-treated CHO (**E**) and LY-B (**F**) cell growth after 72 h in sphingolipid-deficient medium supplemented with sphinganine (seeded cells: 0.25 * 10^6). Significance: (*) $p<0.05$; (**) $p<0.01$ (***) $p<0.001$.

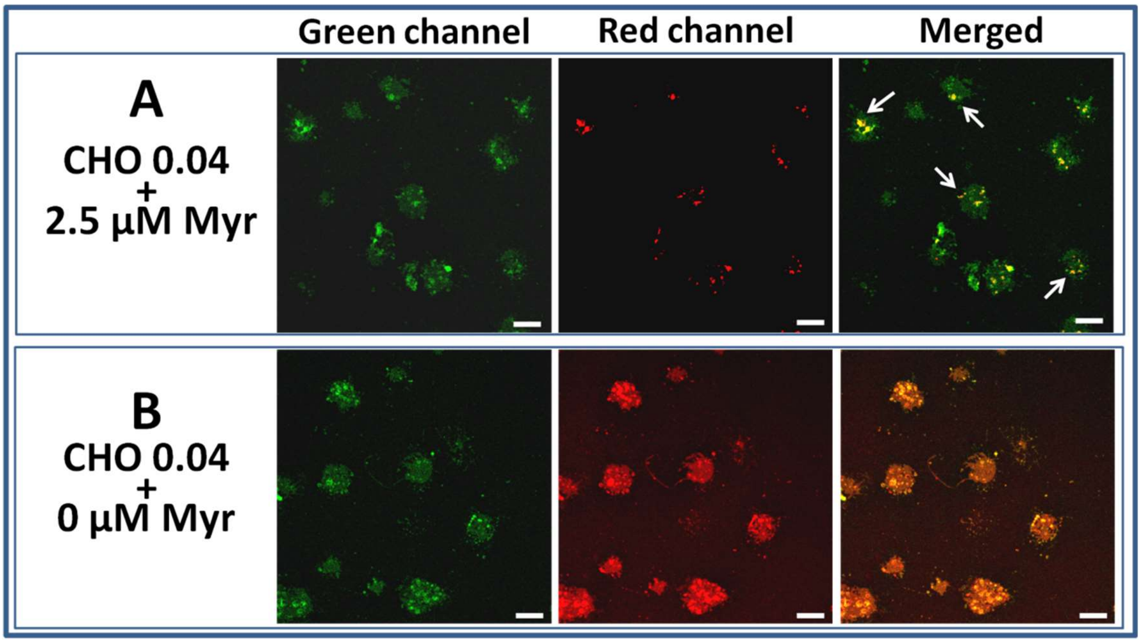

Figure S2. Fluorescence images of PM patches stained with mCherry-lysenin (red). Myriocin treated (**A**) and non-treated CHO cells (**B**) grown in deficient medium. Bar = 50 μm. NBD-PE (green) was used in fluorescence images as a general membrane staining control.

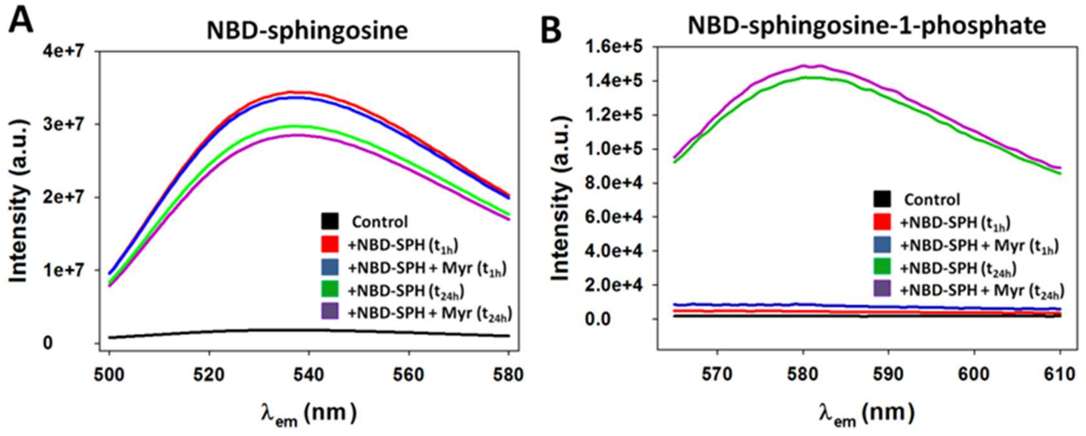

Figure S3. Sphingosine kinase activity in myriocin-treated and non-treated CHO cells: a representative experiment. NBD-sphingosine (A) and NBD-sphingosine-1-phosphate (B) fluorescence emission spectra in CHO cells after addition of 13 µM NBD-sphingosine[13, 65]. Myriocin was added, when required, at a final 2.5 µM concentration. Black lines, control (no NBD-sphingosine); red lines, non-treated cells 1 h after NBD-sphingosine addition; blue lines, myriocin-treated cells 1 h after NBD-sphingosine addition; green lines, non-treated cells 24 h after NBD-sphingosine addition; purple lines, myriocin-treated cells 24 h after NBD-sphingosine addition. Note the different scale in the Y-axes of A and B.

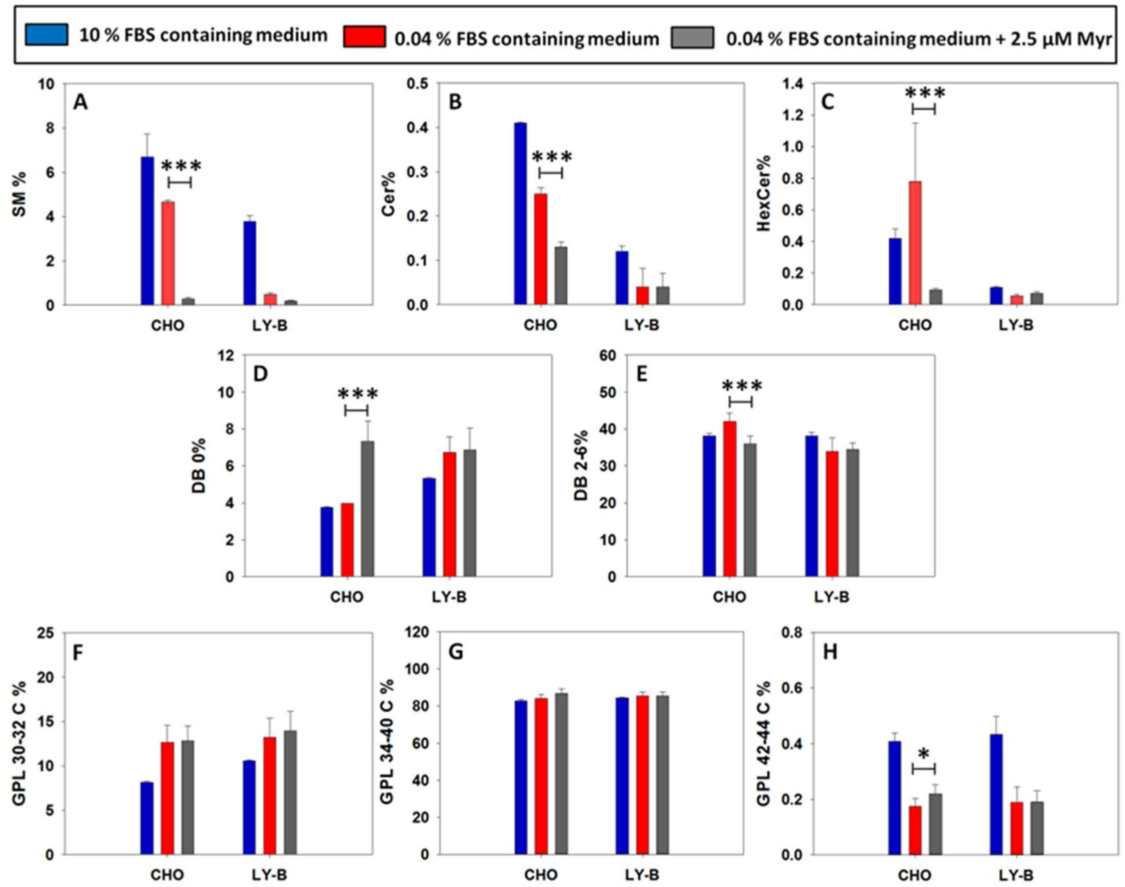

Figure S4. Myriocin treatment effects on the lipid composition of CHO ad LY-B whole cell lipid extracts. Total SM (A), Cer (B) and HexCer (C). Fully saturated (DB = double bond) (D) and polyunsaturated (E) GPL. Short-chain (30-32C) (F), long-chain (34-40C) (G), and very-long chain (42-44C) GPL (H). Only selected lipids are included in the figure, a comprehensive description of the various lipid compositions can be seen in the Supplementary Material Table S1. n=3. Statistical significance was calculated with ANOVA or Student´s t-test, with similar results. Significance: (*) $p<0.05$; (***) $p<0.001$.
